# Supplementary figures and images for: Exploring differentially expressed key genes related to development of follicle by RNA-seq in Peking ducks (Anas Platyrhynchos)
Source: PLoS One. 2019 Jun 25;14(6):e0209061. doi: 10.1371/journal.pone.0209061 (PMC6592512; doi:10.1371/journal.pone.0209061)

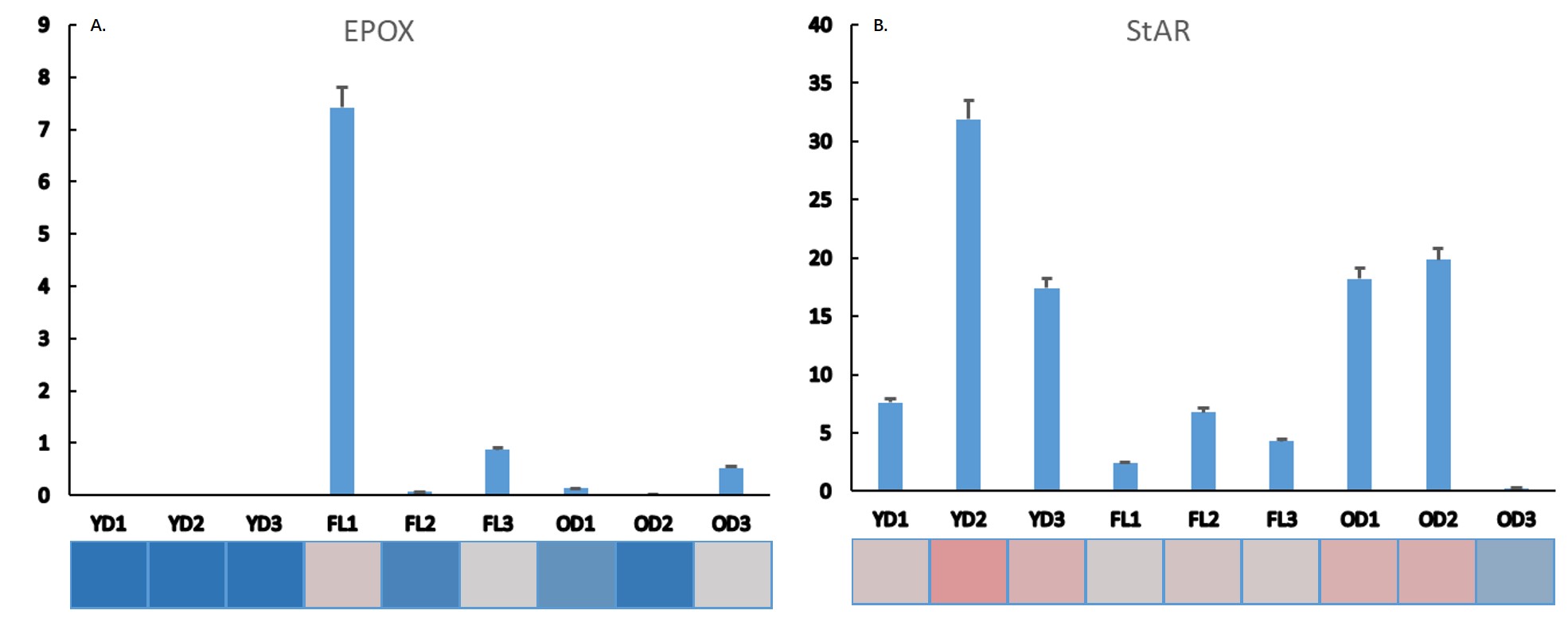

Supplement: S1 Fig — A and B, the expression pattern of EXOP and StAR in bar vs. heatmap generated from its RPKM value. (JPG) [file pone.0209061.s007.jpg]
